# Relationship of intraoperative hypotension with major adverse cardiovascular events and acute kidney injury after pancreaticoduodenectomy

**DOI:** 10.3389/fmed.2026.1754091

**Published:** 2026-03-10

**Authors:** Fan Yu, Yanfei Wei, Jingjiang Huang, Xuechun Chu, Liuyan Wu, Rongting Chen, Xingjun Wang, Youli Hu

**Affiliations:** 1 Department of Anesthesia and Perioperative Medicine, The First Affiliated Hospital of Nanjing Medical University, Nanjing Medical University, Nanjing, China; 2 Department of Anesthesiology, Qilu Hospital (Qingdao), Cheeloo College of Medicine, Shandong University, Qingdao, China

**Keywords:** acute kidney injury, adverse postoperative outcomes, intraoperative hypotension, major adverse cardiovascular events, pancreaticoduodenectomy

## Abstract

**Background:**

Intraoperative hypotension (IOH) is a common concern during major surgery and is associated with end-organ injury. However, its specific impact on major adverse cardiovascular events (MACE) and acute kidney injury (AKI) following pancreaticoduodenectomy (PD) has not been well elucidated.

**Methods:**

A retrospective cohort study was conducted, including 1846 patients who underwent PD between January 2018 and December 2023. Intraoperative mean arterial pressure (MAP) was recorded continuously via radial arterial catheterization. Restricted cubic spline models (RCS) were used to assess the associations of IOH with MACE and AKI. IOH was quantified using four exposure metrics: absolute maximum decrease (AMD), time under threshold (TIME), area under the threshold (AUT), and time-weighted average (TWA) to further analyse the association of MACE and AKI risk at the stratified threshold of MAP <60, 65, 70 mmHg.

**Results:**

Among 1,846 patients enrolled, 211 (11.4%) developed MACE and 52 (2.8%) developed postoperative AKI. Multivariable-adjusted RCS analysis revealed that AKI occurrence increased progressively with decreasing MAP, whereas MACE followed a J-shaped curve with the turn-point of MAP around 65 mmHg. Forest plot analysis found that AMD was the sole metric that maintained a statistically significant association with both MACE and AKI across all tested MAP thresholds (<70, 65, 60 mmHg). Regarding specific thresholds, AMD, AUT, and TWA were significantly associated with MACE at MAP <65 mmHg, whereas AMD, TIME, AUT, and TWA all demonstrated statistical significance for AKI at MAP <60 mmHg.

**Conclusion:**

IOH is associated with MACE and AKI following PD. The higher MAP threshold for MACE (<65 mmHg) than for AKI (<60 mmHg) suggests the need for stricter hemodynamic goals to protect organs with differing ischemic thresholds.

## Introduction

Intraoperative hypotension (IOH) is a common hemodynamic instability during noncardiac surgery that is closely associated with various adverse postoperative outcomes including acute kidney injury (AKI), myocardial injury, mortality, and prolonged length of hospital stay (1–3). The risk of postoperative complications is generally considered as a function of both the severity and the duration of IOH (4–7).

Most of studies frequently characterized IOH as a systolic blood pressure (SBP) <90 mmHg or a mean arterial pressure (MAP) <60 mmHg (8). Alternatively, a reduction of ≥20% from baseline SBP or MAP is also commonly used as a diagnostic threshold (2). Some studies found that intraoperative MAP below 55 mmHg as brief as 1–5 min could potentially enhance the risks of AKI and myocardial injury (1, 6), while MAP below absolute thresholds of 65 mmHg or even 75 mmHg, is also progressively associated with these adverse outcomes (2, 9).

Pancreaticoduodenectomy (PD) as a complex and challenging abdominal operation has a high incidence of IOH following long surgical procedures with extensive digestive tract reconstruction and multiple anastomoses (10, 11). IOH is mainly caused by intraoperative hemorrhage, decreased intravascular volume and reduced peripheral vascular resistance from operational manipulation and surgical stress, which has been considered as a major underlying cause of post-operative complications after PD. However, the relationship between IOH and postoperative complications in PD is still contradictory (12–15). While Ida et al. identified IOH (MAP <65 mmHg) as an independent risk factor for AKI after pancreatic surgery (12), there are other two reports to demonstrate no significant association between IOH and postoperative AKI in PD (13, 14), with one of these two studies reporting that even severe hypotension (MAP <55 mmHg for 10 min) had no significant impact on AKI (14). These conflicting findings make it necessary to further investigate into the potential effect of IOH on the postoperative renal dysfunction after PD. More importantly, the relationship between IOH and postoperative major adverse cardiovascular events (MACE) after PD has not been systematically investigated.

Therefore, we conducted this retrospective cohort analysis to investigate the associations between IOH and postoperative MACE and AKI in patients after PD. In addition to the selected hypotension thresholds, current studies have employed diverse quantification methods to characterize hypotensive exposures, including absolute maximum decrease (AMD), time under threshold (TIME), area under the threshold (AUT) and time-weighted average (TWA). We then employed these four IOH quantification metrics to identify population-specific blood pressure thresholds applicable for the prediction of these adverse outcomes.

## Method

### Study population

**Figure 1:**
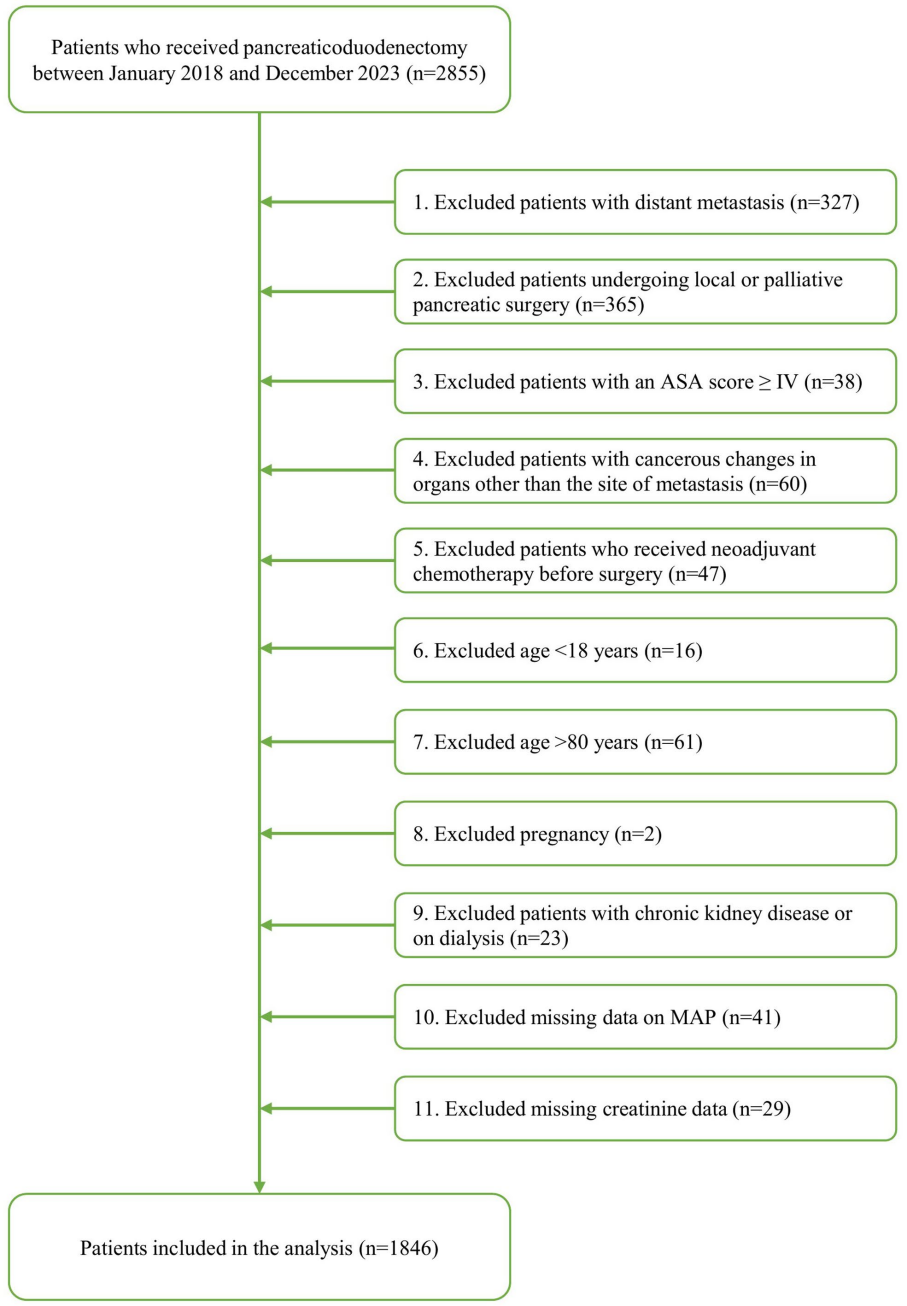
Patient selection flow chart.

We retrospectively analyzed 2,855 patients who underwent PD at the First Affiliated Hospital of Nanjing Medical University between January 2018 and December 2023. After applying inclusion and exclusion criteria, 1,846 patients were included in the final analysis (Figure 1). Eligible patients underwent open PD under general anesthesia, were aged 18–80 years, and were classified as American Society of Anesthesiologists (ASA) physical status I–III.

### Clinical data and indices

Electronic medical records were automatically screened to identify patients who met the inclusion criteria. Subsequently, only data from these eligible patients were extracted for analysis. To mitigate the impact of missing data, a comprehensive manual chart review was performed to abstract and validate supplementary information from the patients’ medical records. The extracted variables included: (1) demographic characteristics (age, sex, BMI); (2) preoperative clinical status (ASA physical status [I–III], comorbidities [hypertension, diabetes mellitus, cardiac disease, ischemic stroke], and medication use [RASI, β-blockers agents, calcium antagonists, diuretics]); (3) intraoperative parameters (surgery duration, total fluid infusion [crystalloids, colloids], blood product administration [red blood cells, plasma], blood loss, total output, urine output, and pancreas texture as hard/median/soft), pathology classified as malignant or benign, and main pancreatic duct size; (4) physiological markers (preoperative and intraoperative blood gas analyses [pH, PaCO₂, HCO₃^−^, lactate, calcium, glucose, hemoglobin]); and (5) postoperative outcomes (length of hospital and ICU stays). Postoperative troponin levels were measured strictly on a clinically indicated basis (e.g., history of cardiovascular disease or abnormal ECG findings), rather than as a routine screening for all patients. The ProAQT system (PULSION, Germany) was applied to constantly monitor hemodynamic parameters, with invasive intraoperative blood pressures were recorded at one-minute intervals.

### Perioperative management

Anesthesia was induced with propofol, remimazolam, sufentanil and rocuronium to facilitate tracheal intubation. Anesthesia was maintained with propofol, remifentanil, rocuronium, dexmedetomidine and sevoflurane. Postoperative analgesia was managed with sufentanil and oxycodone. Standard monitoring included electrocardiography, pulse oximetry, capnography, invasive blood pressure measurements, nasopharyngeal temperature and bispectral index (BIS). Fluid management included crystalloids (Ringer’s lactate or electrolyte solution), colloids (25% albumin or hydroxyethyl starch). Goal-directed fluid therapy (GDFT) was guided by pulse pressure variation (PPV). Vasopressors (Phenylephrine/ Norepinephrine) were initiated after anesthesia induction to maintain MAP at or above 65 mmHg. Packed red blood cells were transfused to maintain hemoglobin levels >8 g/dL.

### Exposure definitions

Baseline MAP was defined as the average of the three MAP readings taken before the induction of anesthesia. Due to the absence of a universally accepted threshold for IOH, we quantified IOH using multiple exposure metrics derived from MAP and performed stratified analyses based on different thresholds. These exposure metrics included:

Lowest MAP: The minimum MAP value recorded during the entire surgical procedure.

TIME: The total duration of time that MAP is below a given threshold, expressed in minutes.

AMD: The maximum decrease in MAP below a given threshold, expressed in mmHg.

AUT: The integrated magnitude and duration of MAP below a given threshold, expressed in mmHg·min.

TWA: AUT for hypotension normalized to the total duration of surgery, expressed in mmHg.

We defined physiologically implausible or abnormal BP values as follows: (1) SBP ≥ 300 mmHg or ≤20 mmHg; (2) SBP ≤ diastolic blood pressure (DBP) + 5 mmHg; (3) DBP ≤ 5 mmHg or ≥225 mmHg; (4) an abrupt SBP change was defined as a fluctuation of ≥80 mmHg in one direction within 1 min, or consecutive fluctuations in opposite directions, each ≥40 mmHg, within 2 min. Additionally, invasive pressure recordings with no change for >5 min were considered indicative of catheter malfunction or blockage and were excluded from analysis (2).

### Outcome definitions

MACE was defined as the occurrence of at least one of the following within 30 days after surgery: (1) acute myocardial injury [high sensitivity cardiac troponin I (hs-cTnI) > 14 ng/L (>99th percentile upper reference limit) without requiring ischemic features] (16); (2) acute myocardial infarction [hs-cTnI elevation with ischemic symptoms and/or electrocardiographic changes and/or new regional wall motion abnormality on imaging] (17); (3) stroke; (4) cardiovascular death; (5) postoperative arrhythmia including ventricular tachycardia, atrial flutter, supraventricular tachycardia and advanced or high-grade AV block (18–20).

The another primary outcome was postoperative AKI, defined using the serum creatinine (SCr) criteria of the kidney disease: Improving Global Outcomes (KDIGO) guidelines: an increase in SCr by ≥0.3 mg/dL (≥26.5 μmol/L) within 48 h or a ≥ 1.5-fold increase from baseline within 7 days (21). The three modified KDIGO stages are: (1) AKI stage 1: SCr 1.5–1.9 times higher than baseline within 7 days, or ≥0.3 mg/dL (≥26.5 umol/L) increase in 48 h or less, (2) AKI stage 2: SCr 2.0 to 2.9 times higher than baseline. (3) AKI stage 3: SCr increase ≥3.0 times baseline, or increase in serum creatinine to ≥4.0 mg/dL (≥353.6 umol/L) or initiation of RRT independent of serum creatinine concentration (22). The urine output criterion was excluded due to the inconsistent documentation of urine volume during the perioperative period.

### Statistical analysis

Patient baseline characteristics and MAP exposures were compared between groups using appropriate statistical tests based on data distribution. Normally distributed continuous variables were expressed as mean ± standard deviation (SD) and analyzed with the Student’s *t* test, whereas non-normally distributed variables were expressed as median (interquartile range, IQR) and analyzed with the Mann–Whitney U test. Categorical variables were presented as percentages (%) and analyzed with χ^2^ or Fisher’s exact tests. Ordered categorical variables were evaluated with the Mann–Whitney U test. Standardized mean differences (SMD) were calculated to assess the balance of baseline characteristics between groups.

The lowest intraoperative blood pressure was defined using a moving average method with a 10-min window. Restricted cubic spline (RCS) regression was employed to model the non-linear relationship between exposure and outcome. We evaluated models with 3, 4, and 5 knots placed at standard percentiles. The optimal model was selected based on the lowest Akaike information criterion (AIC) and Bayesian information criterion (BIC). Models were adjusted for prespecified potential confounders, including: Demographic factors: age, sex, BMI. Comorbidities: hypertension, Ischemic stroke, cardiac diseases, diabetes mellitus. Procedural and clinical factors: ASA physical status, surgery duration, year of surgery, and use of vasoactive agents (norepinephrine/phenylephrine).

Given the absence of a definitive threshold from RCS analyses, stratified analyses were performed based on MAP thresholds. Specifically, separate multivariable models were constructed for each metric (AMD, TIME, AUT, and TWA) to assess their individual associations with postoperative outcomes. A Bonferroni correction was applied to adjust for the four metrics within each outcome, with *p* < 0.0125 (i.e., *p* < 0.05/4 = 0.0125) considered statistically significant, while *p* < 0.05 was considered statistically significant for all other analyses. Multivariable logistic regression models, adjusted for the same confounding variables as the RCS analyses, were used to compute adjusted odds ratios (ORs) with 95% confidence intervals (CIs) for each metric. Patients never experiencing hypotension below the specified threshold served as the reference group.

All statistical analyses were performed using R software (version 4.3.1; R Foundation for Statistical Computing, Vienna, Austria). The rms package was used for RCS modeling and the data.table package for data processing.

## Results

### Baseline characteristics

A cohort of 2,855 patients underwent PD from January 2018 to December 2023. After applying the predefined exclusion criteria, 1846 patients were included in the final analytical cohort (Figure 1). Two hundred and forty-five patients (13.2%) developed at least one major postoperative complication (MACE or AKI). Overall, MACE occurred in 211 patients (11.4%) and AKI occurred in 52 patients (2.8%), with 18 patients (0.9%) developing both complications together. The specific types and characteristics of these complications are detailed in Table 1. Myocardial injury was the most frequent event (*n* = 161), followed by arrhythmia (*n* = 50) in MACE. Among patients with myocardial injury, the median peak troponin level was 21.67 ng/L (IQR: 17.54–34.37). Patients were stratified into groups according to the occurrence of postoperative MACE and AKI (Table 2). Patients in the MACE and AKI groups were significantly older than the control group. Among those who developed postoperative MACE, a significantly higher prevalence of preoperative hypertension, cardiac disease, or ischemic stroke was observed. Postoperative analysis revealed higher lactate levels in the AKI group compared to the controls. Additionally, these patients demonstrated significantly longer operative durations, use of vasoactive drugs and greater fluid replacement requirements. Both MACE and AKI were associated with prolonged postoperative hospital and ICU stays, suggesting that MACE and AKI significantly affect postoperative recovery in patients undergoing PD and increase the likelihood of critical illness.

**Table 1:** Characteristics of the patients with adverse complications.

Complication types	*n* (%)
MACE only	193 (78.8)
AKI only	34 (13.9)
Both MACE and AKI	18 (7.3)
Cardiac complications (*n* = 232)	
Acute myocardial injury	161 (69.4)
Acute myocardial infarction	7 (3.0)
Stroke	2 (0.9)
Cardiovascular death	12 (5.2)
Postoperative arrhythmia	50 (21.6)
Number of cardiac abnormalities (*n* = 211)	
Single event	194 (91.9)
Two events	13 (6.2)
Three events	4 (1.9)
Renal complications (*n* = 52)	
AKI stage 1	38 (73.1)
AKI stage 2	11 (21.2)
AKI stage 3	3 (5.8)

MACE, major adverse cardiovascular events; AKI, acute kidney injury.

**Table 2:** Patient baseline and intraoperative characteristics by postoperative MACE and AKI.

Variable	MACE (*n* = 211)	Non-MACE (*n* = 1,635)	*P* values	SMD	AKI (*n* = 52)	Non-AKI (*n* = 1,794)	*P* values	SMD
Age, y	69 (62–74)	63 (55–69)	<0.001	0.587	68 (63–73)	63 (56–69)	<0.001	0.555
Sex, *n* (%)								
Male	136 (64.5%)	971 (59.4%)	0.179	0.056	41 (78.8%)	1,066 (59.4%)	0.006	0.194
Female	75 (35.5%)	664 (40.6%)			11 (21.2%)	728 (40.6%)		
BMI, kg•mg^−2^	23.3 (21.2–25.4)	22.9 (21.1–24.8)	0.054	0.123	23.5 (21.1–25.8)	22.9 (21.1–24.8)	0.296	0.197
ASA physical status (%)								
I	2 (0.9%)	40 (2.4%)	0.021	0.054	1 (1.9%)	41 (2.3%)	0.008	0.14
II	158 (74.8%)	1,319 (80.6%)			33 (63.5%)	1,444 (80.5%)		
III	51 (24.1%)	276 (16.8%)			18 (34.6%)	309 (17.2%)		
Comorbidity, *n* (%)								
Hypertension	100 (47.4%)	511 (31.3%)	<0.001	0.357	24 (46.2%)	587 (32.7%)	0.051	0.278
Diabetes mellitus	52 (24.6%)	318 (19.4%)	0.082	0.113	12 (23.1%)	358 (20.0%)	0.598	0.076
Cardiac disease	23 (10.9%)	96 (5.9%)	0.010	0.217	5 (9.6%)	114 (6.4%)	0.380	0.121
Ischemic stroke	18 (8.5%)	52 (3.2%)	0.001	0.229	4 (7.7%)	66 (3.7%)	0.132	0.174
Medication use prior to admission, *n* (%)								
RASI	41 (19.4%)	175 (10.7%)	<0.001	0.223	8 (15.4%)	208 (11.6%)	0.381	0.041
β-blocking agents	10 (4.7%)	31 (1.9%)	0.021	0.054	3 (5.8%)	38 (2.1%)	0.106	0.106
Calcium antagonist	50 (23.7%)	254 (15.5%)	0.004	0.124	14 (26.9%)	290 (16.2%)	0.055	0.186
Diuretics	9 (4.3%)	29 (1.8%)	0.033	0.024	2 (3.8%)	36 (2.0%)	0.291	0.199
Vasopressors, *n* (%)								
Phenylephrine	147 (69.7%)	977 (59.8%)	0.006	0.209	44 (84.6%)	1,080 (60.2%)	0.022	0.568
Norepinephrine	24 (11.4%)	84 (5.1%)	<0.001	0.228	7 (13.5%)	101 (5.6%)	0.088	0.269
Year, *n* (%)								
2018–2019	18 (8.5%)	405 (24.8%)	<0.001	0.442	0 (0%)	423 (22.9%)	0.002	0.581
2020–2021	69 (32.7%)	564 (34.5%)			20 (38.5%)	633 (34.3%)		
2022–2023	124 (58.8%)	666 (40.7%)			32 (61.5%)	790 (42.8%)		
Pancreas texture, *n* (%)								
Hard	60 (28.4%)	550 (33.6%)	0.212	0.041	13 (25.0%)	597 (33.3%)	0.343	0.073
Median	97 (46.0%)	656 (40.1%)			26 (50.0%)	727 (40.5%)		
Soft	54 (25.6%)	429 (26.2%)			13 (25.0%)	470 (26.2%)		
Pathology, *n* (%)								
Malignant	179 (84.8%)	1,338 (81.8%)	0.339	0.022	44 (84.6%)	1,473 (82.1%)	0.854	0.021
Benign	32 (15.2%)	297 (18.2%)			8 (15.4%)	321 (17.9%)		
Main pancreatic duct size, *n* (%)								
≤3 mm	116 (55.0%)	990 (60.5%)	0.135	0.04	31 (59.6%)	1,075 (59.9%)	1	0.003
>3 mm	95 (45.0%)	645 (39.5%)			21 (40.4%)	719 (40.1%)		
Preoperative blood gas analysis								
pH	7.46 (7.44–7.48)	7.45 (7.43–7.47)	0.087	0.095	7.46 (7.44–7.48)	7.45 (7.43–7.47)	0.215	0.195
PaCO_2_ (mmHg)	37 (34–40)	38 (35–41)	0.002	0.262	37 (33–39)	38 (35–40)	0.023	0.211
HCO_3_^−^ (mmol/L)	26.6 (26.0–28.2)	26.8 (26.0–28.2)	0.854	0.043	26.8 (25.2–28.0)	26.8 (26.0–28.2)	0.164	0.425
Lac (mmol/L)	0.9 (0.7–1.1)	0.9 (0.7–1.2)	0.644	0.05	1.05 (0.7–1.4)	0.9 (0.7–1.2)	0.041	0.348
Ca^2+^ (mmol/L)	1.13 (1.07–1.16)	1.11 (1.06–1.15)	0.005	0.182	1.10 (1.05–1.15)	1.11 (1.07–1.15)	0.447	0.071
Glu (mmol/L)	6.0 (5.3–7.7)	6.1 (5.3–7.5)	0.948	0.082	6.1 (5.35–8.42)	6.1 (5.3–7.5)	0.664	0.125
Hb (mmol/L)	12.2 (11.1–13.6)	12.6 (11.2–13.9)	0.039	0.152	11.9 (10.9–13.7)	12.5 (11.2–13.9)	0.269	0.144
Surgery time, min	275 (235–338)	275 (230–326)	0.174	0.11	313 (248–353)	273 (230,326)	0.005	0.418
Total infusion, mL	2,800 (2,200–3,450)	2,800 (2,300–3,325)	0.976	0.088	2,900 (2,375–3,825)	2,800 (2,300–3,400)	0.059	0.401
Crystal liquid, mL	1,600 (1,300–2,100)	1,600 (1,500–2,100)	0.550	0.053	1,600 (1,500–2,175)	1,600 (1,500–2,100)	0.040	0.377
Colloidal liquid, mL	1,000 (700–1,200)	1,000 (700–1,200)	0.972	0.019	1,000 (700–1,200)	1,000 (700–1,200)	0.998	0.143
Red blood cell infusion, U	0 (0–2)	0 (0–2)	0.023	0.125	0 (0–3)	0 (0–2)	0.006	0.352
Plasma, mL	0 (0–350)	0 (0–300)	0.015	0.16	225 (0–381)	0 (0–300)	0.002	0.402
Total output, mL	850 (550–1,250)	800 (550–1,200)	0.249	0.097	800 (500–1,100)	800 (550–1,200)	0.695	0.078
Blood loss, mL	300 (200–500)	300 (200–500)	0.816	0.106	352 (200–525)	300 (200–500)	0.230	0.265
Urine volume, mL	450 (300–700)	400 (300–650)	0.046	0.11	350 (300–612)	400 (300–700)	0.449	0.029
Intraoperative blood gas analysis								
pH	7.39 (7.36–7.41)	7.39 (7.36–7.42)	0.317	0.065	7.38 (7.33–7.40)	7.39 (7.36–7.42)	0.016	0.384
PaCO_2_ (mmHg)	41 (38–44)	41 (38–43)	0.260	0.055	41.5 (39.75–44)	41 (38–44)	0.140	0.009
HCO_3_^−^ (mmol/L)	24.9 (24.3–25.5)	24.9 (24–25.7)	0.909	0.014	24.9 (24.4–25.5)	24.9 (24.1–25.7)	0.798	0.128
Lac (mmol/L)	1 (0.7–1.2)	1 (0.8–1.2)	0.381	0.009	1.1 (0.9–1.4)	1 (0.8–1.2)	0.009	0.386
Ca^2+^ (mmol/L)	1.06 (1.04–1.1)	1.06 (1.03–1.1)	0.608	0.018	1.06 (1.01–1.09)	1.06 (1.03–1.11)	0.215	0.096
Glu (mmol/L)	8.7 (7.5–10.0)	8.7 (7.5–9.8)	0.876	0.023	8.4 (7.15–9.4)	8.7 (7.5–9.8)	0.211	0.048
Hb (mmol/L)	11 (10.1–11.9)	11 (10.2–12.2)	0.123	0.115	11 (10.1–12.6)	11 (10.2–11.9)	0.969	0.039
Length of hospital stay, d	27 (20–38)	21 (16–28)	<0.001	0.485	28 (21–39)	21 (16–29)	<0.001	0.546
Length of ICU stay, d	0 (0–4)	0 (0–0)	<0.001	0.321	0 (0–6)	0 (0–0)	<0.001	0.44

Continuous variables are presented as median (25th, 75th percentiles), categorical variables as *n* (%). *P* values from the Wilcoxon rank sum test for continuous variables and χ^2^ test for categorical variables. Standardized mean differences (SMD) were calculated to assess the balance of baseline characteristics between groups.

SMD, standard mean difference; BMI, body mass index; ASA, American Society of Anesthesiologists; RASI, renin–angiotensin system inhibitors; pH, arterial plasma pH; PaCO₂, arterial carbon dioxide partial pressure; HCO₃⁻, plasma bicarbonate ions; Hb, arterial hemoglobin; Lac, arterial plasma lactate; Ca^2^⁺, arterial plasma calcium ions; MACE, major adverse cardiovascular events; AKI, acute kidney injury.

### Moving average curve analysis

**Figure 2:**
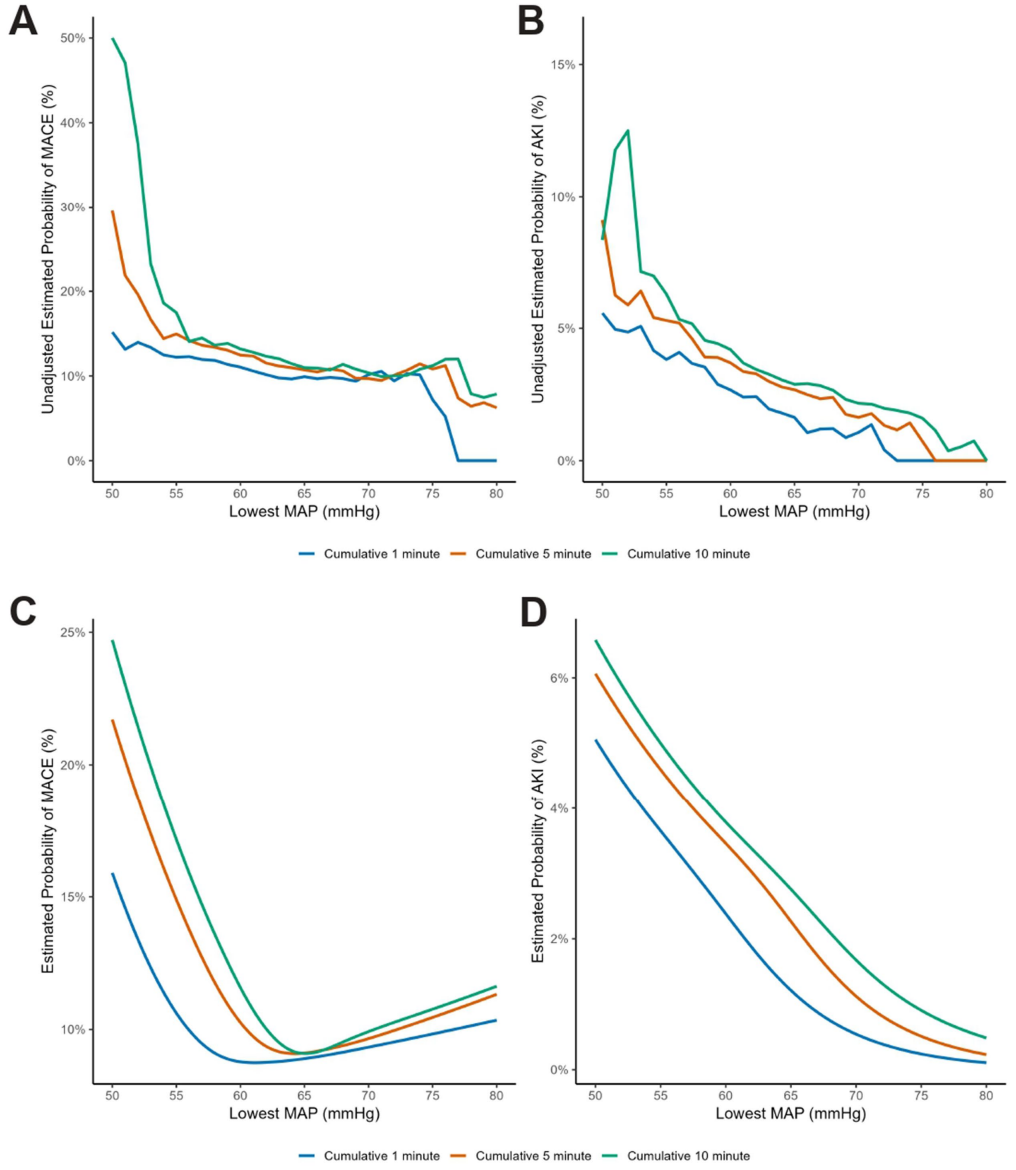
The lowest MAP thresholds for MACE and AKI. Univariable and multivariable relationship between outcomes and absolute lowest MAP thresholds. **(A,B)** Estimated probability of MACE and AKI from univariable moving-window analysis with the width of 10% data; **(C,D)** were from multivariable logistic regression smoothed by restricted cubic spline with 3 knots at 10th, 50th, and 90th percentiles of lowest MAP.

The moving average curve clearly demonstrated an inverse correlation between the unadjusted estimated probabilities of both postoperative MACE and AKI and the intraoperative lowest MAP (Figures 2A,B). Specifically, as the lowest MAP decreased, the risks of MACE and AKI exhibited a progressively escalating trend. This association became more pronounced with prolonged durations of hypotension (from cumulative ≥1 min to ≥10 min), manifested by a steeper slope of the risk curves, indicating a progressively intensifying strength of correlation.

### Multivariable-adjusted RCS curve analysis

RCS is a valuable tool for perioperative research, offering an interpretable analysis by flexibly capturing complex, non-linear relationships between continuous variables and outcomes. Based on the minimization of AIC and BIC values, a model with 3 knots was selected as the optimal fit for the analysis (Supplementary Table S1). The RCS regression revealed divergent risk patterns between postoperative MACE and AKI in relation to the lowest MAP (Figures 2C,D).

The risk of AKI demonstrated a clear and consistent dose–response relationship with both the severity and duration of hypotension. Across the entire observed range, the risk of AKI monotonically increased as MAP decreased. Furthermore, the risk curves were distinctly stratified by the cumulative duration of hypotension (10 min > 5 min > 1 min) without any intersection. These distinct curves indicate that at any given MAP level, a longer hypotensive exposure was invariably associated with a correspondingly higher risk of AKI.

In contrast, the relationship for MACE was more complex presenting with a J-shaped curve. A key feature for MACE was the intersection of the duration-based curves near the lowest point of this J-shaped curve, at a MAP of approximately 65 mmHg. This turn point within the low risk range suggests the influence of hypotensive duration on MACE could be minimized at MAP around 65 mmHg. As MAP progressively reduced below this point, MACE occurrence increased dramatically, while higher MAP above 65 mmHg started to be related to a mild increase for MACE risk. To address the potential heterogeneity within the composite MACE outcome, we performed a sensitivity analysis to evaluate the association between the lowest MAP and individual components. The RCS analysis restricted to myocardial injury demonstrated a robust J-shaped association, mirroring the pattern observed in the primary composite outcome (Supplementary Figure S1).

### Intraoperative hemodynamic comparisons

This analysis was to precisely quantify the magnitude of the association between hypotensive exposure below predefined, commonly utilized clinical thresholds and the risk of MACE and AKI.

Univariate analysis of intraoperative hemodynamic parameters revealed a significant association between hypotension and the incidence of postoperative MACE and AKI (Table 3). Patients who developed AKI exhibited significantly lowest MAP values sustained for ≥1 min (mean ± SD: 55.38 ± 5.17 mmHg vs. median [IQR]: 59.00 [55.00–63.00] mmHg in the No AKI group; *p* < 0.001) and ≥5 min (61.40 ± 4.67 mmHg vs. 63.80 [60.20–67.00] mmHg; *p* = 0.002). Similarly, the MACE group showed a significantly lowest MAP for ≥1 min (57.00 [54.00–62.00] mmHg vs. 59.00 [55.00–63.00] mmHg; *p* = 0.002) and ≥5 min (mean ± SD: 62.64 ± 5.99 mmHg vs. median [IQR]: 63.80 [60.40–67.00] mmHg; *p* = 0.033). Notably, patients with AKI consistently exhibited lowest MAP at a lower level compared to those with MACE.

**Table 3:** Univariable relationship between MAP exposures and outcomes.

Variable	Overall	MACE (*n* = 211)	Non-MACE (*n* = 1,635)	*P* values	AKI (*n* = 52)	Non-AKI (*n* = 1,794)	*P* values
Baseline MAP, mmHg	89.00 (80.33–99.00)	90.00 (81.00–100.50)	89.00 (80.33–98.67)	0.209	93.55 ± 14.52	89.00 (80.33–98.67)	0.071
Lowest MAP ≥1 min, mmHg	59.00 (55.00–63.00)	57.00 (54.00–62.00)	59.00 (55.00–63.00)	0.002	55.38 ± 5.17	59.00 (55.00–63.00)	<0.001
Lowest MAP ≥5 min, mmHg	63.60 (60.20–67.00)	62.64 ± 5.99	63.80 (60.40–67.00)	0.033	61.40 ± 4.67	63.80 (60.20–67.00)	0.002
Lowest MAP ≥10 min, mmHg	66.20 (62.82–69.68)	65.59 ± 5.70	66.20 (63.00–69.70)	0.134	64.89 ± 4.62	66.20 (63.00–69.70)	0.051
TIME <55 mmHg, min	0.00 (0.00–0.00)	0.00 (0.00–1.00)	0.00 (0.00–0.00)	0.003	0.00 (0.00–2.00)	0.00 (0.00–0.00)	<0.001
AUT <55 mmHg·min	0.00 (0.00–0.00)	0.00 (0.00–1.50)	0.00 (0.00–0.00)	0.003	0.00 (0.00–4.25)	0.00 (0.00–0.00)	<0.001
TWA <55 mmHg	0.00 (0.00–0.00)	0.00 (0.00–0.00)	0.00 (0.00–0.00)	0.003	0.00 (0.00–0.01)	0.00 (0.00–0.00)	<0.001
TIME <60 mmHg, min	1.00 (0.00–4.00)	2.00 (0.00–6.00)	1.00 (0.00–4.00)	<0.001	3.00 (1.00–7.50)	1.00 (0.00–4.00)	<0.001
AUT <60 mmHg·min	1.00 (0.00–12.00)	4.00 (0.00–20.00)	1.00 (0.00–11.00)	<0.001	9.50 (2.00–28.25)	1.00 (0.00–12.00)	<0.001
TWA <60 mmHg	0.00 (0.00–0.03)	0.01 (0.00–0.05)	0.00 (0.00–0.03)	<0.001	0.03 (0.01–0.07)	0.00 (0.00–0.03)	<0.001
TIME <65 mmHg, min	8.00 (2.00–20.00)	9.00 (2.00–25.50)	7.00 (2.00–20.00)	0.108	10.50 (4.75–26.25)	7.00 (2.00–20.00)	0.021
AUT <65 mmHg·min	23.00 (4.00–74.00)	33.00 (7.00–96.50)	23.00 (4.00–72.00)	0.02	57.00 (19.25–106.25)	23.00 (4.00–72.00)	<0.001
TWA <65 mmHg	0.06 (0.01–0.19)	0.09 (0.02–0.25)	0.06 (0.01–0.18)	0.022	0.13 (0.05–0.29)	0.06 (0.01–0.19)	0.002
TIME <70 mmHg, min	32.50 (12.00–68.00)	31.00 (11.00–75.00)	33.00 (13.00–67.00)	0.881	36.00 (15.25–84.00)	32.00 (12.00–67.75)	0.152
AUT <70 mmHg·min	130.50 (45.00–304.75)	152.00 (43.50–364.00)	128.00 (46.00–297.00)	0.268	173.00 (89.50–378.50)	129.00 (45.00–302.75)	0.015
TWA <70 mmHg	0.35 (0.12–0.78)	0.37 (0.11–0.91)	0.34 (0.12–0.77)	0.308	0.46 (0.21–0.90)	0.34 (0.12–0.78)	0.038
TIME <75 mmHg, min	90.00 (46.00–148.00)	90.00 (38.00–148.00)	90.00 (47.00–148.00)	0.303	105.50 (52.75–176.75)	90.00 (45.25–148.00)	0.171
AUT <75 mmHg·min	454.00 (207.25–884.75)	472.00 (173.00–969.50)	451.00 (213.50–871.50)	0.91	500.00 (290.75–1011.25)	454.00 (205.25–873.50)	0.081
TWA <75 mmHg	1.23 (0.56–2.30)	1.16 (0.46–2.37)	1.23 (0.58–2.29)	0.976	1.30 (0.76–2.63)	1.23 (0.56–2.29)	0.166

Continuous variables are presented as mean ± SD or median (25th, 75th percentiles), categorical variables as *n* (%). *P* values from the Student’s *t* test or Wilcoxon rank sum test for continuous variables and χ^2^ test for categorical variables, as appropriate.

MAP, mean arterial pressure; AUT, area under the threshold; TWA, time-weighted average; MACE, major adverse cardiovascular events; AKI, acute kidney injury.

Regarding cumulative hypotension, patients with MACE or AKI demonstrated a significantly greater hypotensive burden at MAP thresholds of <55 mmHg and <60 mmHg across all metrics (TIME, AUT, TWA) compared to patients without these outcomes (all *p* ≤ 0.003). At a MAP <65 mmHg, the association remained significant for AKI across TIME (*p* = 0.021), AUT (*p* < 0.001), and TWA (*p* = 0.002). For MACE, however, only AUT (*p* = 0.020) and TWA (*p* = 0.022) were significant, while TIME was not (*p* = 0.108). At the MAP <70 mmHg threshold, the association with AKI persisted only for AUT (*p* = 0.015) and TWA (*p* = 0.038), while no metrics were significant for MACE. No significant associations were observed for either MACE or AKI at the MAP <75 mmHg threshold. Crucially, when comparing the two complication groups, patients with AKI consistently demonstrated a substantially greater hypotensive burden across all these metrics than patients who experienced MACE. For instance, the median duration of MAP <65 mmHg was 10.5 min for the AKI group but 9 min for the MACE group, and the area under the threshold (MAP<65 mmHg) was 57 mmHg·min for the AKI group compared to 33 mmHg·min for the MACE group. This indicates that the magnitude and duration of intraoperative hypotension were most pronounced in patients who subsequently developed AKI than MACE.

### Multivariable-adjusted stratified analysis

**Figure 3:**
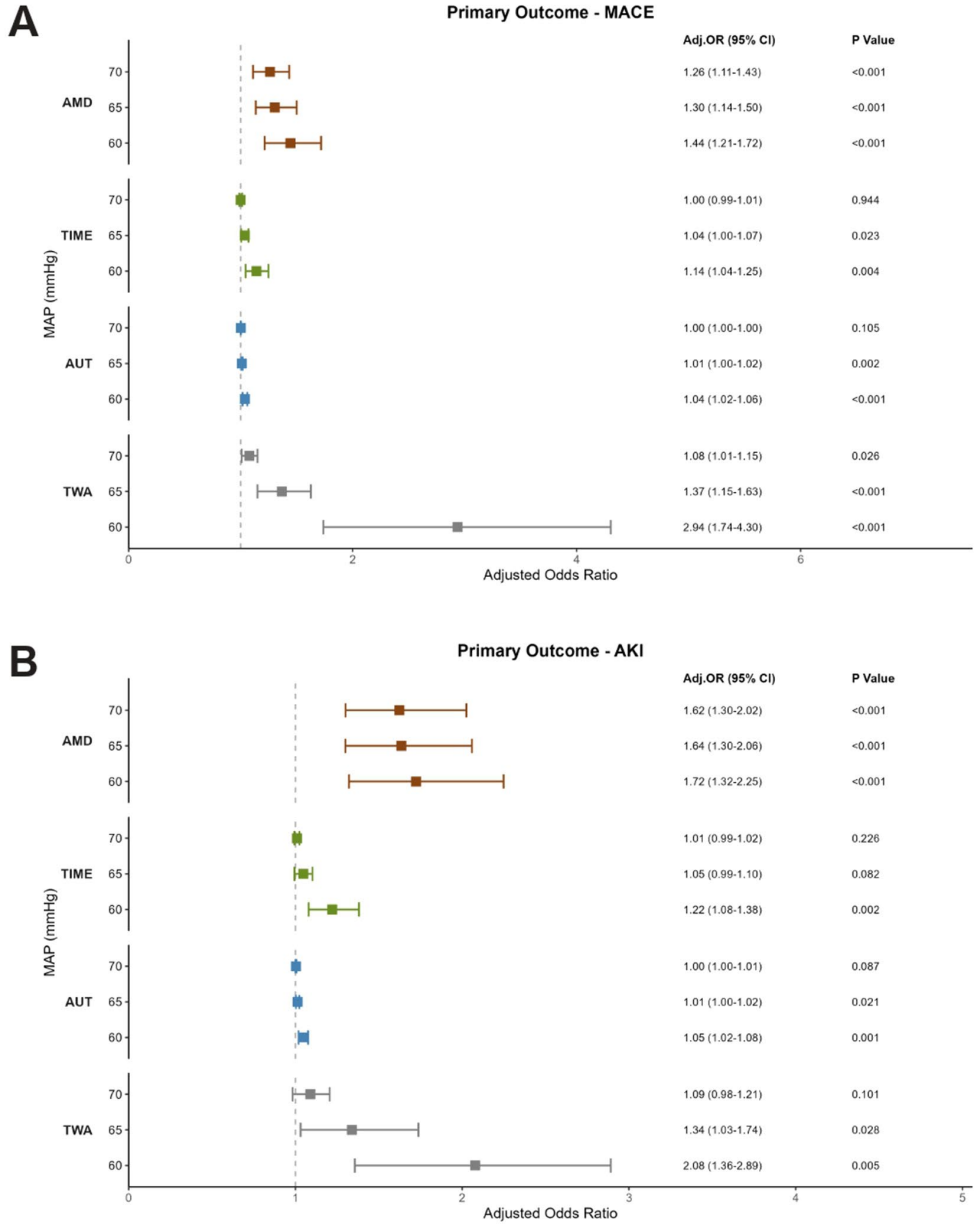
Adjusted odds ratios and 95% confidence intervals (CIs) for adverse outcomes based on different intraoperative MAP exposure metrics. **(A)** Forest plot for MACE from the adjusted analysis. **(B)** Forest plot for AKI from the adjusted analysis. For both outcomes, the models are based on the following MAP exposure metrics, shown in order: AMD (absolute maximum decrease), TIME (time spent under the absolute threshold), AUT (area under the threshold), and TWA (time-weighted average).

An assessment of the forest plots revealed the following significant commonalities in the association between IOH and both MACE and AKI (Figure 3). A clear inverse dose–response relationship was observed, whereby the risk of both outcomes progressively increased with decreasing MAP thresholds. While AMD was the sole metric that maintained a statistically significant association with both MACE and AKI across all tested MAP thresholds (<70, 65, and 60 mmHg), the other metrics (TIME, AUT, and TWA) exhibited a threshold-dependent pattern, becoming statistically significant primarily at lower MAP thresholds (<65 and 60 mmHg). This indicated that the “depth” of hypotension, irrespective of its thresholds, constituted an independent and fundamental risk factor.

For AKI, at the threshold of MAP <60 mmHg, all four hypotension metrics (AMD, TIME, AUT, and TWA) demonstrated a statistically significant association with AKI. When the MAP threshold was elevated to 65 and 70 mmHg, the association with cumulative metrics (TIME, AUT, TWA) was no longer statistically significant. These findings remained robust in sensitivity analyses after adjustment for confounding factors (Supplementary Figure S2). For MACE, at the threshold of MAP <65 mmHg, AMD, AUT, TWA remained strongly associated with the risk of MACE, but TIME did not show statistical significance. This observation aligned with the RCS analysis, where the cumulative time curves intersected at this specific value. This convergence suggests that the influence of hypotension duration on MACE is minimized when MAP is maintained around 65 mmHg. To further validate these findings, we performed an additional sensitivity analysis excluding data from the COVID-19 period (2020–2021). The results did not significantly change in the sensitivity analysis excluding data from 2020 to 2021 (Supplementary Figure S3).

## Discussion

This retrospective cohort study included 1846 patients underwent PD at the First Affiliated Hospital of Nanjing Medical University. We systematically evaluated associations between IOH and postoperative MACE and AKI and found a dose-dependent relationship of these two complications with hypotension severity by multivariate analysis.

Significant association of IOH with the risk of MACE and AKI has been well reported after noncardiac surgery based on various IOH diagnostic thresholds of MAP, SBP, DBP and pulse pressure. Some studies found that IOH exhibited significant associations with MACE and AKI after noncardiac surgery at all evaluated MAP thresholds (<75, 65, and 55 mmHg), with progressively stronger associations at lower thresholds (2, 3). Based on two prospective cohort studies, IOH defined as SBP <90 mmHg for a cumulative duration ≥ 10 min was independently associated with both MACE and AKI, regardless of preexisting coronary artery disease severity (23, 24). DBP <60 mmHg linked to the postoperative complications is also reported (25). However, pulse pressure was a weaker predictor of postoperative myocardial and renal injury compared to steady-state blood pressure components (MAP, SBP, DBP) (5). In the present study, we first applied RCS to find association of IOH with MACE and AKI, and then further use four exposure metrics, including AMD, TIME, AUT and TWA to accurately measure potential IOH threshold on MACE and AKI. These metrics have been validated in previous studies for their accuracy in integrating both the magnitude and duration of hypotensive insults to quantify cumulative hypotensive exposure (6, 26, 27) The depth-weighted lower blood pressure area was significantly associated with myocardial injury and mortality, whereas the duration-weighted variable showed no significant association, indicating that depth of hypotension contributes more than duration to adverse outcomes (28). This is consistent with our findings that AMD demonstrated superior predictive value compared to TIME, AUT and TWA (6).

Our findings align with previous large-scale studies demonstrating that MAP less than 60 mmHg is independently associated with postoperative AKI (29, 30). Similarly, a multicenter cohort study demonstrated that IOH (MAP ≤ 55 mmHg) was significantly associated with persistent acute kidney disease (AKD), defined as AKI persisting beyond 7 days postoperatively, but not with delayed AKD occurring between 8 and 90 days after surgery across any MAP thresholds examined (≤75, ≤65, or ≤55 mmHg) (27). In our study, we observed a clear dose–response relationship at the MAP <60 mmHg threshold.

Our observation of a J-shaped relationship between intraoperative MAP and MACE and the multivariate logistic regression further illustrated progressively narrowing confidence intervals and diminishing odds ratios for most exposure metrics as thresholds increased from 60 to 70 mmHg. This result establishes 65 mmHg as a clinically meaningful turn point for MACE and a MAP range as MACE risk increased not only with profound hypotension (e.g., <65 mmHg) but also at relatively higher MAP levels. Our findings are consistent with other published reports. The BP-CARES trial demonstrated no incremental benefit in reducing cardiovascular events with an intensive MAP target (≥80 mmHg) compared with a conventional target (≥65 mmHg) (31). In addition, another PRETREAT randomized clinical trial found that a risk-stratified blood pressure (≥70, 80, 90 mmHg) had no obvious improvement on postoperative outcomes as compared with usual care targeting ≥65 mmHg (32). Similarly, in the multi-trial IMPROVE study, an individualized perioperative blood pressure management strategy (target MAP 65–110 mmHg) did not reduce adverse outcomes compared to routine management (target MAP ≥ 65 mmHg) in patients undergoing major abdominal surgery (33). These findings along with the present study underscore the crucial MAP threshold above 65 mmHg during PD and suggest that simply raising blood pressure targets may not improve postoperative outcomes.

In the present study, the absence of cardiovascular benefit at higher MAP thresholds may be attributable to increased catecholamine exposure, as more phenylephrine/norepherine requirement for the treatment of hypotension could induce myocardial depression with increased afterload, arrhythmias, and increased metabolic demand (34, 35), thereby offsetting the potential advantages of maintaining higher perfusion pressures. Furthermore, clinicians tend to positively treat hypotension in high-risk patients (e.g., those with advanced age or cardiac comorbidities) to maintain higher MAP targets. Consequently, these vulnerable patients are exposed to higher cumulative doses of vasopressors. Therefore, the present study provides evidence for the clinical practice of the non-cardiac major surgery that it is crucial to keep intraoperative and postoperative MAP at an appropriated level so that the patients could have more benefit for the cardiac function and avoid severe postoperative outcomes such as MACE.

Our findings are consistent with differential autoregulatory thresholds (36). Organ-specific differences in autoregulatory capacity may explain the differential sensitivity to IOH observed between MACE and AKI. The kidney possesses robust intrinsic autoregulatory mechanisms that maintain relatively constant renal blood flow (RBF) and glomerular filtration rate (GFR) over a perfusion pressure range of 80–180 mmHg through myogenic responses and tubuloglomerular feedback (37, 38). In contrast, the heart lacks true pressure-dependent autoregulation. Coronary blood flow is primarily governed by metabolic demand, with adenosine, nitric oxide, and K_ATP channels mediating vasodilation to match oxygen supply to demand (39, 40). These physiological differences may explain our finding that MACE demonstrated greater sensitivity to moderate hypotension (MAP <65 mmHg) than AKI.

However, two recent single-center studies failed to find clear associations between IOH and postoperative AKI after PD (13, 14). Lydon et al. analyzed 200 patients with only 20 AKI events (10% incidence), potentially resulting in insufficient power to detect associations between IOH and AKI. The small number of events may have limited the statistical power of the analysis (14). In another study, Gu et al. examined a larger cohort of 844 patients with 98 AKI cases (11.6% incidence) and employed RCS analysis for threshold identification (13). They employed 5-min intervals for blood pressure monitoring, which may not capture brief but severe hypotensive episodes. Furthermore, there were two basic metrics for IOH quantification in that study using the lowest intraoperative blood pressure and cumulative time below thresholds (SBP < 100 mmHg and DBP < 60 mmHg). In the present study, we established a comprehensive quantification system for IOH encompassing four core metrics: AMD, TIME, AUT, and TWA. This metric integration provides a more complete risk assessment than the isolated measurements used previously.

While this study provides insights into the association between IOH and postoperative AKI/MACE in pancreatic surgery, several limitations should be acknowledged. First, as a single-center retrospective analysis, our findings may be influenced by specific institutional practices and inherent selection biases. Consequently, external validation across diverse multi-center cohorts is imperative before these results can be translated into changes in clinical guidelines. Second, our AKI definition relied solely on serum creatinine criteria due to unavailable urine output data, potentially underestimating the true AKI incidence. This kind of underestimation, combined with the overall low number of recorded AKI events, directly resulted in limited statistical power. However, given that hypotension is a key driver of the low urine output, missing these cases likely weakened our results, this suggests the real link between IOH and AKI is actually stronger than what we found. Third, as a retrospective study, residual confounding may exist due to unmeasured intraoperative factors. Although we adjusted for a comprehensive set of potential confounders, other specific intraoperative parameters such as anesthetic depth and continuous cardiac output were not available for analysis. Finally, our study defined intraoperative hypotension solely based on absolute MAP thresholds. We did not analyze relative hypotension (e.g., percentage decrease from preoperative baseline). Future studies should consider incorporating relative thresholds to further clarify the association of IOH with MACE and AKI.

## Conclusion

Our study demonstrated significant associations between IOH and both postoperative MACE and AKI after PD. Our analysis indicated that AKI was associated with MAP <60 mmHg, while MACE was associated with a threshold of MAP <65 mmHg. Among IOH metrics, AMD maintained statistical significance across all MAP thresholds for both outcomes. These findings highlight the critical importance of both hypotension depth and duration in postoperative cardiovascular and renal dysfunction, providing clinical evidence for the clinicians to keep close monitoring and management of perioperative hemodynamic stability, especially during major non-cardiac surgery.

## Data Availability

The data analyzed in this study is subject to the following licenses/restrictions: Data supporting this study’s findings can be obtained upon request from the corresponding author and are not publicly accessible due to privacy and ethical considerations. Requests to access these datasets should be directed to Fan Yu, 2849265691@qq.com.

## Supplementary material

Supplementary Figure S1 The lowest MAP thresholds for myocardial injury. The association was modeled using a multivariable logistic regression smoothed by restricted cubic spline with 3 knots at 10th, 50th, and 90th percentiles of lowest MAP.

Supplementary Figure S2 Adjusted odds ratios and 95% Confidence Intervals (CIs) for AKI based on different intraoperative MAP exposure metrics in sensitivity analysis. Confounding factors: age, hypertension, vasopressors and year of surgery.

Supplementary Figure S3 Adjusted odds ratios and 95% Confidence Intervals (CIs) for adverse outcomes based on different intraoperative MAP exposure metrics after excluding data from the COVID-19 period (2020–2021). **(A)** Forest plot for MACE from the adjusted analysis. **(B)** Forest plot for AKI from the adjusted analysis.
