# Supplementary material for: Relationship of intraoperative hypotension with major adverse cardiovascular events and acute kidney injury after pancreaticoduodenectomy
Source: Front Med (Lausanne). 2026 Mar 10;13:1754091. doi: 10.3389/fmed.2026.1754091 (PMC13008874; doi:10.3389/fmed.2026.1754091)
Supplement: Supplementary file 1 [file Table_1.docx]

**Table S1** Selection of Optimal Knots for Restricted Cubic Spline Analysis

| Outcome | Duration, min | Number of Knots | Knot Percentiles | AIC | BIC | ΔAIC | ΔBIC |
| --- | --- | --- | --- | --- | --- | --- | --- |
| AKI | 1 | 3 | 10%, 50%, 90% | 443.53 | 520.8 | 0 | 0 |
| AKI | 1 | 4 | 5%, 35%, 65%, 95% | 445.31 | 528.1 | 1.78 | 7.3 |
| AKI | 1 | 5 | 5%, 35%, 50%,65%,95% | 447.18 | 535.48 | 3.65 | 14.68 |
| AKI | 5 | 3 | 10%, 50%, 90% | 448.26 | 525.46 | 0 | 0 |
| AKI | 5 | 4 | 5%, 35%, 65%, 95% | 449.84 | 532.55 | 1.58 | 7.09 |
| AKI | 5 | 5 | 5%, 35%, 50%,65%,95% | 451.16 | 539.38 | 2.9 | 13.93 |
| AKI | 10 | 3 | 10%, 50%, 90% | 451.13 | 528.3 | 0 | 0 |
| AKI | 10 | 4 | 5%, 35%, 65%, 95% | 452.37 | 535.04 | 1.23 | 6.75 |
| AKI | 10 | 5 | 5%, 35%, 50%,65%,95% | 452.41 | 540.59 | 1.28 | 12.3 |
| MACE | 1 | 3 | 10%, 50%, 90% | 1235.35 | 1312.62 | 0 | 0 |
| MACE | 1 | 4 | 5%, 35%, 65%, 95% | 1236.9 | 1319.69 | 1.55 | 7.07 |
| MACE | 1 | 5 | 5%, 35%, 50%,65%,95% | 1238.68 | 1326.99 | 3.33 | 14.37 |
| MACE | 5 | 3 | 10%, 50%, 90% | 1239.54 | 1316.74 | 0 | 0 |
| MACE | 5 | 4 | 5%, 35%, 65%, 95% | 1241.47 | 1324.19 | 1.93 | 7.45 |
| MACE | 5 | 5 | 5%, 35%, 50%,65%,95% | 1242.82 | 1331.05 | 3.28 | 14.31 |
| MACE | 10 | 3 | 10%, 50%, 90% | 1240.26 | 1317.42 | 0 | 0 |
| MACE | 10 | 4 | 5%, 35%, 65%, 95% | 1242.04 | 1324.71 | 1.78 | 7.29 |
| MACE | 10 | 5 | 5%, 35%, 50%,65%,95% | 1242.05 | 1330.24 | 1.79 | 12.82 |

Abbreviations: MACE = major adverse cardiovascular events; AKI = acute kidney injury; AIC= akaike information criterion; BIC= bayesian information criterion (BIC).
