# Supplementary figures and images for: Relationship of intraoperative hypotension with major adverse cardiovascular events and acute kidney injury after pancreaticoduodenectomy
Source: Front Med (Lausanne). 2026 Mar 10;13:1754091. doi: 10.3389/fmed.2026.1754091 (PMC13008874; doi:10.3389/fmed.2026.1754091)

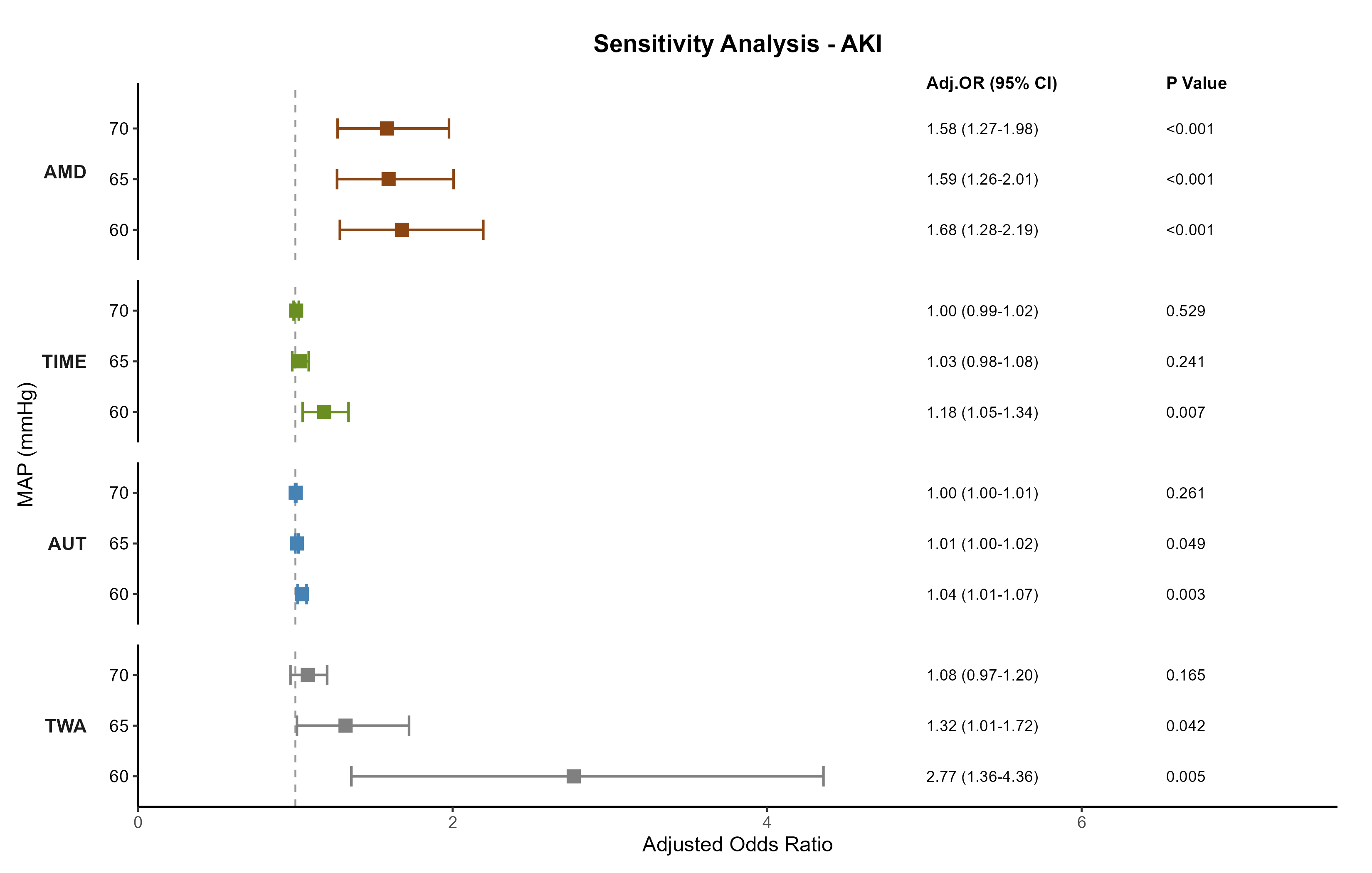

Supplement: Supplementary file 2 [file Image_1.png]

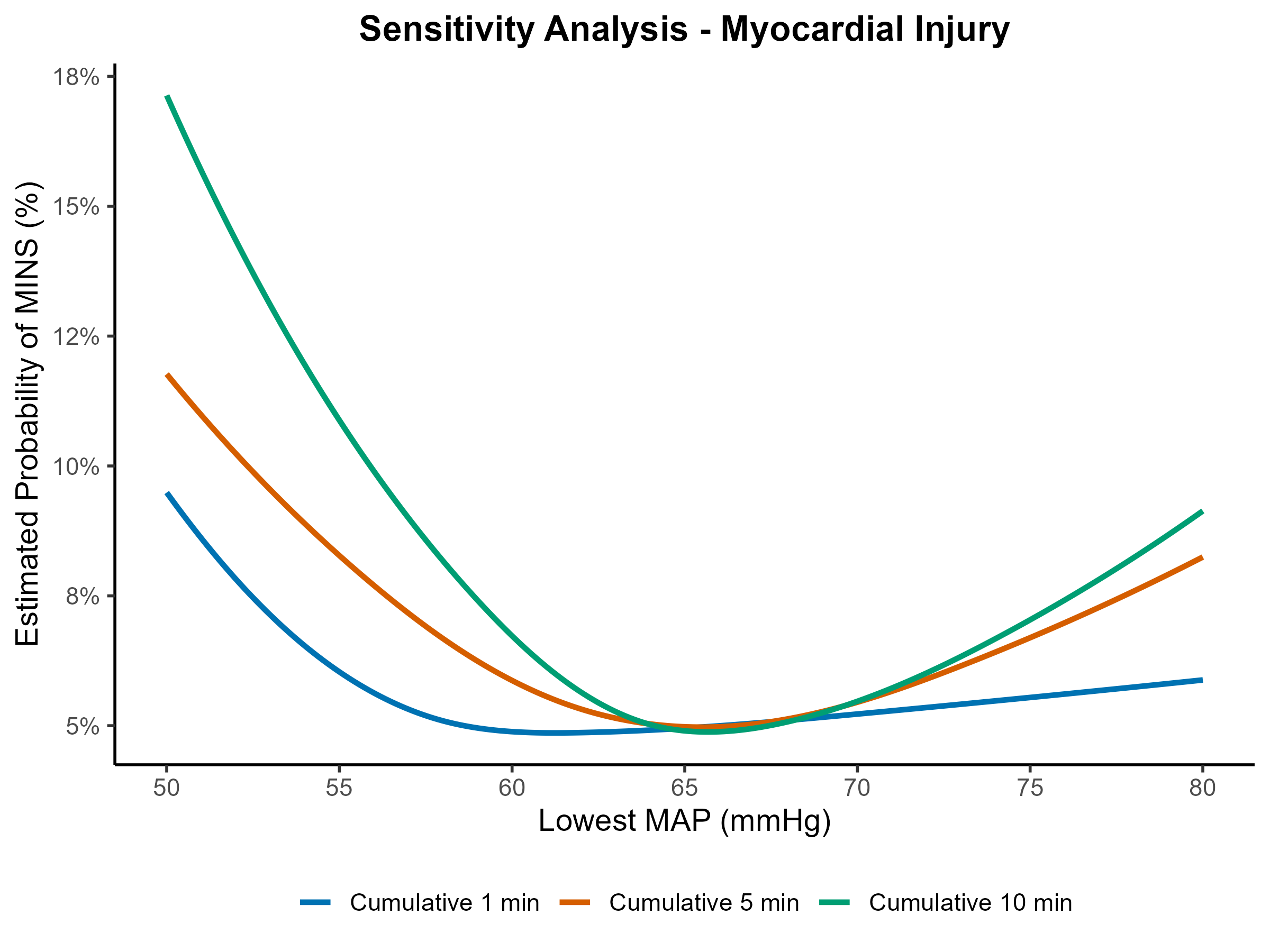

Supplement: Supplementary file 3 [file Image_2.png]

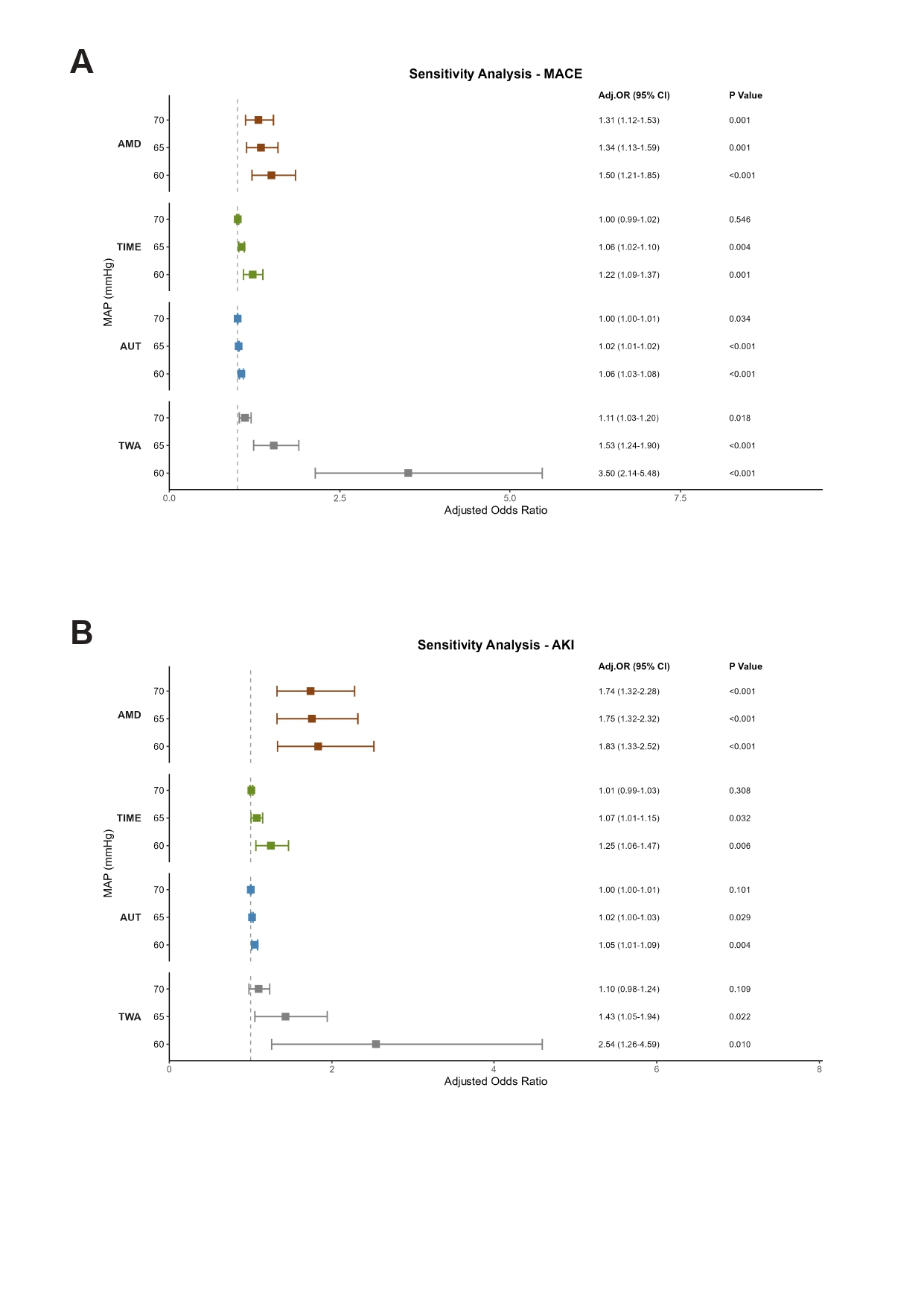

Supplement: Supplementary file 4 [file Image_3.jpeg]
